# Programmatic outcomes of adolescents in differentiated service delivery models in South Africa

**DOI:** 10.4102/sajid.v40i1.733

**Published:** 2025-07-16

**Authors:** Phumzile M. Shaku, Kate Rees, Barry Mutasa, Christina Maluleke, Steven Mashele, Christine Njuguna

**Affiliations:** 1 Anova Health Institute, Tzaneen, South Africa; 2 Anova Health Institute, Johannesburg, South Africa; 3 Department of Community Health, School of Public Health, University of the Witwatersrand, Johannesburg, South Africa; 4 Anova Health Institute, Polokwane, South Africa; 5 Department of Health, Mopani District, Giyani, South Africa

**Keywords:** adolescents, differentiated service delivery models, HIV, retention, viral suppression, outcomes

## Abstract

**Background:**

Adolescents living with HIV face barriers that impede adherence and retention. Differentiated service delivery (DSD) models aim to improve retention and viral suppression (VS), but there is limited programmatic evidence from South Africa on DSD outcomes.

**Objectives:**

This study aimed to measure 12 month retention and VS proportions in adolescents enrolled in DSD and clinic-based care, and measure the association between 12 month retention, VS and covariates.

**Method:**

A retrospective cohort study was conducted in the Mopani District, Limpopo province, using TIER.Net data. The study included adolescents aged 10–19 years enrolled in DSD between 01 September 2019 and 30 September 2022, and those eligible for DSD with viral load < 50 copies/mL. The study measured 12-month retention and VS proportions. Multivariable logistic regression measured association among 12-month retention, VS and exposure variables.

**Results:**

A total of 646 adolescents in DSD and 1282 in clinic-based care were included. Twelve-month retention was 92.7% (599/646) in DSD and 89.0% (1141/1282) in clinic-based care. There was no association between 12-month retention and being enrolled in DSD versus clinic-based care. Twelve-month VS (< 50 copies/mL) was 63.5% (251/395) in DSD, compared to clinic-based care 51.0% (494/969). In multivariable regression, being on DSD was associated with higher VS at < 50 copies/mL (Adjusted Odds Ratio [AOR] 1.6; 95% confidence interval: 1.2–2.1; *p* < 0.001) than clinic-based care.

**Conclusion:**

Differentiated service delivery improved VS in adolescents in a rural setting and should be prioritised to improve outcomes.

**Contribution:**

Differentiated service delivery improves adolescent VS in a rural setting.

## Introduction

South Africa has the largest antiretroviral therapy (ART) programme globally, with approximately 7.6 million people living with HIV and 5.6 million receiving ART.^1^ In 2022, estimates from the Thembisa Model reported an overall HIV prevalence of 12.6%, including 1.2% among children aged 0–14 years and 6.0% among adolescents and youth aged 15–24 years.^2^

South Africa aims to meet the Joint United Nations Programme on HIV/AIDS (UNAIDS) 95-95-95 targets by 2030.^3^ However, the HIV care cascade for children and adolescents under 15 years is lagging, with 81% diagnosed, 71% on ART and 59% being virally suppressed.^3^

Access to ART for adolescents living with HIV is hindered by barriers such as frequent clinic visits, unstructured lives and long service waiting times.^4^ Non-disclosure and delayed disclosure of HIV status are major barriers to adherence.^5^ Anticipated stigma and fear of HIV status discovery may prevent clinic attendance, affecting retention.^6^ To improve retention, the World Health Organization (WHO) expanded differentiated service delivery (DSD) eligibility from 2017 to include adolescents and children as young as 2 years old.^7^ Differentiated service delivery is a client-centred approach that tailors and streamlines HIV services to the needs and circumstances of People Living with HIV (PLHIV), reducing clinic visits and offering community-based services to improve outcomes such as viral suppression (VS) and retention.^8,9^

In this study, DSD refers to differentiated ART delivery for stable adolescents as outlined in the South African adherence guidelines for HIV, TB and non-communicable diseases.^10^ Stable adolescents on DSD receive a repeat prescription and treatment from the Central Chronic Dispensing and Distribution programme (CCMDD).^10^ Since 2016, South Africa adopted DSD for stable adults over 18 years on ART and other chronic medications.^11^ In 2020, with updates in 2023, eligibility was expanded to include children and adolescents who met specific criteria^10,12^: (1) aged five to 18 years; (2) no regimen change or dosage change in the past 3 months; (3) viral load (VL) in the past 12-months < 50 copies/mL; (4) disclosure to the adolescent initiated by the caregiver; (5) no other conditions requiring continuous clinical care. The adolescent or caregiver in the case of younger adolescents voluntarily opts and provides consent for enrolment after receiving information about the models.^10^

In Mopani District, the following DSD models are implemented: facility-based pick-up points, where clients collect pre-dispensed medication from a designated area; pharmacy fast-lane, where collection of pre-packed medicine parcels is fast-tracked within the facility; external pick-up points, involving the collection of medication from an external service provider; and adherence clubs, which offer group-format services within the facility, with facilitators monitoring client adherence and wellness.^10^ In all DSD models, a 2–3-month supply of pre-packed ART is provided and clinical visits are scheduled every 6 months.

Fifty-eight facilities in Mopani District offer youth-friendly services through Youth Care Clubs (YCC), which provide integrated HIV clinical care, pre-packed ART, psychosocial support and comprehensive sexual and reproductive health services to adolescents.^13^ The YCC model includes adolescents and youths living with HIV who are newly initiated on ART as well as those on ART with suppressed or unsuppressed VLs, to foster peer learning. Once adolescents are stable and meet the eligibility criteria, they are enrolled in one of the three DSD models and receive pre-packed medicine parcels from CCMDD within the YCCs.

Differentiated service delivery has improved retention and VS among adolescents and youth in Sub-Saharan Africa.^14^ A systematic review found youth-friendly clinics and community-based ART were effective in improving VS and retention in those aged 10–24 years.^14^ In Lesotho, districts with DSD models, such as teenage clubs, community adherence clubs and peer support groups, had higher retention rates (73%) compared to those without (63%).^15^ A South African study reported a 94.3% retention rate at 12 months among adolescents and youth aged 12–25 years in DSD models.^16^

However, evidence on the effectiveness of scaled-up DSD models for adolescents aged 10–19 years in South Africa, particularly in rural areas like Mopani District in Limpopo province, is limited. There is also a scarcity of information on retention and VS across different service delivery modalities. This study aimed to: (1) assess the proportion of adolescents sustaining VS and retention at 12 months in DSD models compared to clinic-based care; (2) evaluate the association between demographic and clinical characteristics for 12-month retention and VS.

## Research methods and design

### Study design

We conducted a cross-sectional study using routinely collected programmatic data from TIER.Net, an electronic register containing clinical and demographic information of people with HIV and tuberculosis.

### Study setting

The study was conducted in Mopani District, in the north-eastern part of the Limpopo province. Limpopo province has an estimated population of 6 572 721.^17^ The Mopani District with five sub-districts: Ba-Phalaborwa, Greater Giyani, Greater Letaba, Greater Tzaneen and Maruleng, has a population of 1 372 873.^17.18^ The HIV prevalence in the Limpopo province is 8.9% translating to an estimated 570 000 people.^19^ There are 104 primary healthcare facilities, 3 non-medical sites and 8 hospitals providing HIV treatment and care for adolescents.^18^ The study included 113 facilities providing DSD.

### Study population

#### Inclusion criteria

This study included two groups of adolescents aged 10–19 years: (1) enrolled in DSD models between 01 September 2019 and 30 September 2022; or (2) receiving clinic-based care defined as receiving routine HIV care comprising frequent clinic visits, with at least one documented VL < 50 copies/mL between 01 September 2019 and 30 September 2022 and potentially eligible for DSD. Reasons for not enrolling adolescents in clinic-based care included missed opportunities where the clinician did not notice eligibility, failure to meet eligibility criteria, such as being pregnant or lacking a valid identity document, or the client’s preference for clinic-based care.

#### Exclusion criteria

The study excluded adolescents who: (1) were lost to follow-up, died or transferred out before 01 September 2019; (2) enrolled in DSD or had their first VL < 50 copies/mL from 01 October 2022; (3) were in clinic-based care and did not have at least one VL < 50 copies/mL between 01 September 2019 and 30 September 2022.

### Sampling

TIER.Net was used as the sampling frame. Demographic and clinical data were extracted for adolescents aged 10–19 years who met the inclusion criteria from 113 health facilities.

### Study variables

For adolescents to achieve and maintain VS, they must be consistently retained in care. The study focused on two outcomes:

1. Twelve-month retention in care, defined as still in care for 365 days or more after DSD enrolment date or eligibility date. Those transferring out to another clinic, died or lost to follow-up before 365 days were classified as not retained. Lost to follow-up was defined as clients who missed a clinic appointment by ≥ 12 weeks without known cause, death or transfer out. Transfer out referred to clients who moved their ART care to another facility.
2. Viral suppression for 12 months is defined as a VL between 8 and 20 months after DSD enrolment date (DSD group) or eligibility date for adolescents in clinic-based care. This wide VL window was selected because of insufficient VLs taken at 12 months for the analysis. In this study, a < 50 copies/mL cut-off was considered VS.^20^ An additional VL cut-off of < 1000 copies/mL was also reported for VS as per the 95-95-95 UNAIDS VS targets.^21^ Viral suppression proportions were calculated as total virally suppressed divided by total number of clients with VLs.
  **Exposure variables:** Demographic variables included gender, age at last visit and sub-district. Clinical variables included dates of VL collection and VL values. Treatment variables included ART start dates, ART regimen at last visit, DSD enrolment date, DSD model, treatment month, treatment outcome and treatment outcome date.

### Data analysis

Prior to data analysis, all identifying information were removed. Descriptive statistics were used to describe client characteristics, with continuous variables analysed using mean and standard deviation for normally distributed data, medians and interquartile ranges for skewed data. Categorical variables were described using proportions.

Simple proportions measured the 12-month retention and VS for two cut-offs (< 50 copies/mL and < 1000 copies/mL) in DSD models and clinic-based care. Differences were assessed using Chi-squared Statistics or Fisher’s exact tests.

Multivariable logistic regression was conducted to measure association between outcomes (12-month retention and 12-month VS), and the following exposure variables: study group, age at last visit, gender, ART duration, ART regimen type (first or second-line ART), ART regimen backbone and sub-district.

Antiretroviral therapy duration was defined from ART start date to DSD enrolment date or eligibility date (clinic-based care group). Exposure variables were pre-selected based on known associations with the outcome variables and availability on TIER.Net database. Multivariable Logistic Regression Analysis reported adjusted odds ratios (AOR), 95% confidence intervals (CIs) and *p*-values (< 0.05 considered significant). Data were analysed using STATA software Version 18.^22^

### Ethical considerations

Ethical approval was obtained from the Human Sciences Research Council’s Research Ethics Committee (REC 3/22/08/18). This study used de-identified TIER.Net data which are routinely collected at healthcare facilities for monitoring purposes, therefore individual consent was not required.

## Results

A total of 1935 adolescents met the inclusion criteria. Of these, 1285 (66.5%) and 650 (33.5%) were in the clinic-based care and DSD groups, respectively. Seven adolescents were excluded for death, loss to follow-up or transferred out on or before the DSD enrolment or eligibility date. The final sample included 1928 individuals (*n* = 646 DSD group and *n* = 1282 clinic-based care group).

### Characteristics of adolescents in differentiated service delivery and clinic-based care groups

Of the 1928 adolescents, 646 (33.5%) were in DSD models and 1282 (66.5%) in the clinic-based care group (Table 1). For the DSD models (*n* = 646), 26 (4.0%) were in adherence clubs, 239 (37.0%) in external pick-up points and 381 (59.0%) in facility pick-up points. Almost 75%, (*n* = 475) in DSD were older, aged between 15–19 years (*p* < 0.001) and 365 (56.5%) were female (*p* = 0.879). Most adolescents in DSD had been on ART for more than 5 years; ranging from 69.2% to 77.8% across the models, compared to clinic-based care clients (58.1%) (Table 1).

**TABLE 1:** Characteristics of adolescents enrolled in differentiated service delivery compared to those in clinic-based care.

Variable	Clinic-based care (*n* = 1282)†		DSD model (*n* = 646)		*p*	Adherence clubs (*n* = 26)		External pick-up point (*N* = 239)		Facility pick-up point (*N* = 381)		*p*
	*N*	%	*N*	%		*N*	%	*N*	%	*N*	%	
**Age (years)**												
10–14	602	47.0	171	26.5	0.000	7	26.9	69	28.9	95	24.9	0.557
15–19	680	53.0	475	73.5	-	19	73.1	170	71.1	286	75.1	-
**Gender**												
Female	729	56.9	365	56.5	0.879	15	57.7	136	56.9	214	56.2	0.976
Male	553	43.1	281	43.5	-	11	42.3	103	43.1	167	43.8	-
**ART duration (years)**												
< 1	144	11.3	18	2.8	0.000	2	7.7	9	3.7	7	1.8	0.188
1–5	390	30.6	138	21.4	-	6	23.1	44	18.4	88	23.1	-
> 5	739	58.1	490	75.9	-	18	69.2	186	77.8	286	75.1	-
**ART regimen type** ‡												
First-line ART	1220	95.4	620	96.0	0.553	26	100.0	228	95.4	366	96.1	0.521
Second-line ART	59	4.6	26	4.0	-	0	-	11	4.6	15	3.9	-
**ART regimen backbone** §												
Efavirenz or Nevirapine-based regimen	309	24.2	86	13.3	0.000	4	15.4	34	14.2	48	12.6	0.316
Dolutegravir-based regimen	803	62.8	517	80.0	-	21	80.8	183	76.6	313	82.2	-
Lopinavir-based regimen or ritonavir-based regimen	167	13.1	43	6.7	-	1	3.9	22	9.2	20	5.3	-
**Sub-district**												
Ba-Phalaborwa	189	14.7	111	17.2	0.000	18	69.2	36	15.1	57	15.0	0.000
Greater Giyani	308	24.0	98	15.2	-	4	15.4	25	10.5	69	18.1	-
Greater Letaba	200	15.6	146	22.6	-	0	0.0	50	20.9	96	25.2	-
Greater Tzaneen	503	39.2	232	35.9	-	2	7.7	116	48.5	114	29.9	-
Maruleng	82	6.4	59	9.1	-	2	7.7	12	5.0	45	11.8	-

ART, antiretroviral therapy; DSD Model, differentiated service delivery model.

† , Excludes nine clients with missing information on ART duration;

‡ , Clinic-based care group (*n* = 1279) excludes three clients with missing information on ART regimen type;

§ , Clinic-based care group (*n* = 1279) excludes three clients with missing information on ART regimen backbone.

Most adolescents were on first-line ART, 96.0% for DSD and 95.4% for clinic-based care (*p* = 0.553). More adolescents in DSD were on dolutegravir-based regimen at the last clinic visit 517 (80.0%), compared to 803 (62.8%) in clinic-based care. Just over a third of adolescents in DSD (35.9%) and clinic-based care (39.2%) were enrolled in Greater Tzaneen sub-district. The exception was for the adherence clubs, where 69.2% (18/26) were enrolled in Ba-Phalaborwa sub-district (Table 1).

### Twelve-month retention for adolescents in differentiated service delivery group and clinic-based care

At the end of the study period, 81.7% (1576/1928) adolescents remained in care, 5.5% (106/1928) were lost to follow-up, 0.9% (18/1928) had died and 11.8% (228/1928) had transferred out (Data not shown).

**FIGURE 1:**
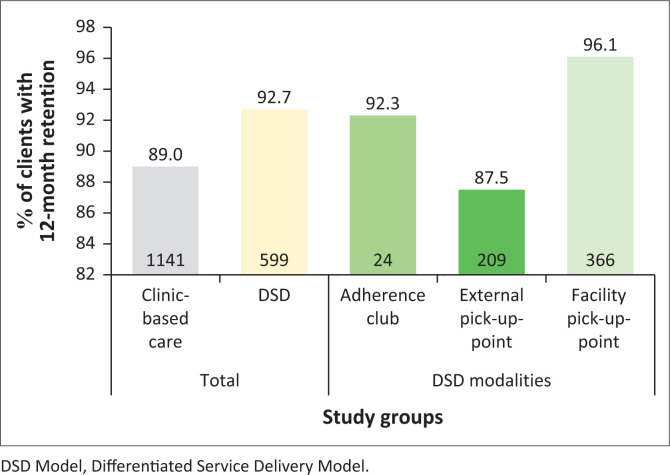
Twelve-month retention on antiretroviral therapy comparing differentiated service delivery to clinic-based care, and differentiated service delivery modalities.

Overall, 12-month retention on ART was comparable; 92.7% (95% CI: 90.4–94.6; 599/646) for the DSD group versus 89.0% (95% CI: 87.2–90.7; 1141/1282) for clinic-based group (proportion difference = 3.7%, *p* = 0.009) as shown in Figure 1 and mentioned in Table 2.

**TABLE 2:** Twelve-month retention for adolescents enrolled in differentiated service delivery compared to those in clinic-based care.

Study group	Twelve-month retention percentage, *n* (%)		95% Confidence interval	Percentage difference	*p*
	*n*	%			
Total (*n* = 1928)	1740	90.3	88.84–91.54	N/A	N/A
DSD (*n* = 646)	599	92.7	90.44–94.61	3.7%	0.009
Clinic-based care (*n* = 1282)	1141	89.0	87.16–90.66	-	-

DSD Model, Differentiated Service Delivery Model; N/A, not applicable.

Within the DSD modalities (*n* = 646), facility pick-up points had the highest 12-month ART retention at 96.1% (366/381), compared to 92.3% (24/26) for adherence clubs and 87.5% (209/239) for external pick-up-points as shown in Figure 1.

### Factors associated with 12 months antiretroviral therapy retention in the total study population

In Multivariable Logistic Regression Analysis, there was no difference between DSD (AOR = 1.1; 95% CI: 0.7–1.7; *p* = 0.621) versus clinic-based care (Table 3). Factors associated with increased odds of 12-month retention were: being on ART for 1–5 years (AOR = 4.0; 95% CI: 2.4–6.7; *p* < 0.001), and > 5 years (AOR = 5.2; 95% CI: 3.2–8.5; *p* < 0.001) compared to below 1 year, being on a dolutegravir-based ART regimen, (AOR = 7.4; 95% CI: 5.0–10.9; *p* = 0.010), and a lopinavir/ritonavir-based ART regimen (AOR = 2.5; 95% CI: 1.3–4.8; *p* = 0.005) compared to an efavirenz/nevirapine-based ART regimen, and being enrolled within Giyani sub-district compared to Ba-Phalaborwa (AOR = 2.3; 95% CI: 1.2–4.4; *p* = 0.010) (Table 3).

**TABLE 3:** Logistic regression for factors associated with 12-month retention on differentiated service delivery versus clinic-based care.

Variable	12 months retention DSD versus clinic-based care (*n* = 1928)†					
	Unadjusted odds ratio	95% CI	*p*	Adjusted odds ratio	95% CI	*p*
**Study group**						
Clinic-based care	Ref	-	-	Ref	-	-
DSD	1.57	1.12–2.22	0.010	1.11	0.74–1.65	0.621
**Age (years)**						
10–14	Ref	-	-	Ref	-	-
15–19	0.66	0.48–0.91	0.011	0.55	0.37–0.80	0.002
**Gender**						
Female	Ref	-	-	Ref	-	-
Male	1.46	1.07–2.00	0.018	1.27	0.89–1.81	0.181
**ART duration (years)**						
< 1	Ref	-	-	Ref	-	-
1–5	3.88	2.47–6.08	0.000	4.02	2.42–6.69	0.000
> 5	4.96	3.32–7.42	0.000	5.21	3.19–8.52	0.000
**ART regimen type**						
First-line	Ref	-	-	Ref	-	-
Second-line	0.34	0.20–0.59	0.000	0.33	0.16–0.68	0.003
**ART regimen backbone**						
Efavirenz or Nevirapine	Ref	-	-	Ref	-	-
Dolutegravir	5.60	3.99–7.86	0.000	7.36	4.99–10.85	0.010
Lopinavir or Ritonavir	2.03	1.27–3.24	0.003	2.51	1.31–4.79	0.005
**Sub-district**						
Ba-Phalaborwa	Ref	-	-	Ref	-	-
Greater Giyani	1.33	0.74–2.37	0.342	2.31	1.22–4.35	0.010
Greater Letaba	0.55	0.33–0.93	0.025	1.03	0.58–1.85	0.910
Greater Tzaneen	0.88	0.54–1.44	0.612	1.24	0.73–2.11	0.430
Maruleng	0.38	0.21–0.70	0.002	0.52	0.27–1.01	0.052

ART, antiretroviral therapy; CI, Confidence Interval; DSD Model, Differentiated Service Delivery Model; Ref, reference group.

† , Excludes nine clients with missing information on ART duration.

Older age (15–19 years) was associated with decreased odds of 12-month ART retention (AOR = 0.6; 95% CI: 0.4–0.8; *p* = 0.002) compared to adolescents aged 10–14 years (Table 3).

### Factors associated with 12-month retention for differentiated service delivery group only (*n* = 646)

In multivariable logistic regression, there was no association between DSD Models and 12-month retention as shown in Online Appendix 1 Table 1-A1.

### Viral suppression for adolescents in differentiated service delivery and clinic-based care groups

Overall, 70.7% (1364/1928) of adolescents had a VL done during the follow-up period. Of these, 54.6% (745/1364) had VS < 50 copies/mL at 12 months (data not shown).

Among adolescents in the DSD group 61.2% (395/646) had a 12-month VL done compared to 75.6% (969/1282) in the clinic-based group (Table 4).

**TABLE 4:** Twelve-month viral load suppression (< 50 copies/mL and < 1000 copies/mL) for differentiated service delivery modalities compared to clinic-based care group.

Study group	Total VLs done		*p*	Viral suppression%†	Viral suppression‡		95% CI	*p*
	*n*	%			*n*	%		
**VL < 50 copies/mL**								
Clinic-based care (*n* = 1282)	969	75.6	0.000	38.5	494	51.0	47.78–54.17	0.000
DSD (*n* = 646)	395	61.2	-	38.9	251	63.5	58.58–68.30	-
**DSD Models**								
Adherence club (*n* = 26)	21	80.8	0.087	42.3	11	52.4	29.78–74.29	0.507
External pick-up points (*n* = 239)	140	58.6	-	36.8	88	62.9	54.29–70.87	-
Facility-based pick-up points (*n* = 381)	234	61.4	-	39.9	152	65.0	58.47–71.06	-
**VL < 1000 copies/mL**								
Clinic-based care (*n* = 1282)	969	75.6	0.000	64.4	825	85.1	82.74–87.32	0.000
DSD (*n* = 646)	395	61.2	-	57.1	369	93.4	90.50–95.66	-
**DSD Models**								
Adherence club (*n* = 26)	21	80.8	0.087	76.9	20	95.2	76.18–99.88	0.275
External pick-up points (*n* = 239)	140	58.6	-	53.1	127	90.7	84.64–94.96	-
Facility-based pick-up points (*n* = 381)	234	61.4	-	58.3	222	94.9	91.21–97.32	-

VL, viral load; CI, Confidence Interval; DSD Model, Differentiated Service Delivery Model.

† , Viral suppression, as proportion of all adolescents;

‡ , Viral suppression, as proportion of those with a VL done.

Twelve-month VS (< 50 copies/mL) was 63.5% (95% CI: 58.6–68.3; 251/395) for DSD group, compared to 51.0% (95% CI: 47.8–54.2; 494/969) in the clinic-based care group (proportion difference: 12.5%, *p* < 0.001) (Table 4).

Twelve-month VS (< 1000 copies/mL) was 93.4% (95% CI: 90.5–95.7; 369/395) for those in DSD group, compared to 85.1% (95% CI: 82.7–87.3; 825/969) in clinic-based care (proportion difference = 8.3%; *p* < 0.001) (Table 4).

Twelve-month VLs were done in 80.8% (21/26) of adolescents in adherence clubs, 58.6% (140/239) in external pick-up points and 61.4% (234/381) in facility pick-up points (Table 4).

Twelve-month suppression (< 50 copies/mL) was lowest for adherence clubs 52.4%, compared to 65.0% for facility pick-up points and 62.9% for external pick-up points. At 12 months, VS at < 1000 copies/mL was similar for adherence clubs and facility pick-up points (95.0%) compared to 90.7% for external pick-up points (Table 4).

Low-level viremia (50–999 copies/mL) was observed in 32.9% (449/1364) of the cohort. Adolescents in clinic-based care showed a higher proportion of low-level viremia, with 34.2% (331/969) compared to 29.9% (118/395) in DSD (Data not shown).

### Factors associated with 12-month viral suppression

Being enrolled in DSD versus clinic-based care increased the odds of 12-month VS (< 50 copies/ml) after adjusting for age at last clinic visit, gender, ART duration, ART regimen type, ART regimen backbone and sub-district, (AOR = 1.6 95% CI 1.2–2.1; *p* < 0.001). No other variables were significantly associated with VS < 50 copies/mL (Table 5).

**TABLE 5:** Multivariable logistic regression for factors associated with 12-month viral load suppression at < 50 copies/mL (*n* = 1360) and < 1000 copies/mL cut-off (*n* = 1360).

Variable	VL suppression (< 50 copies/mL)						VL suppression (< 1000 copies/mL)					
	Unadjusted odds ratios	95% CI	*P*	Adjusted odds ratio	95% CI	*P*	Unadjusted odds ratio	95% CI	*P*	Adjusted odds ratio	95% CI	*P*
**Study group**												
Clinic-based care	Ref	-	-	Ref	-	-	Ref	-	-	Ref	-	-
DSD	1.68	1.32–2.13	0.000	1.60	1.24–2.07	0.000	2.48	1.60–3.83	0.000	2.20	1.39–3.48	0.001
**Current age (years)**												
10	Ref	-	-	Ref	-	-	Ref	-	-	Ref	-	-
15–19	1.08	0.87–1.34	0.493	0.94	0.74–1.19	0.582	0.99	0.71–1.36	0.930	0.82	0.57–1.18	0.295
**Gender**												
Female	Ref	-	-	Ref	-	-	Ref	-	-	Ref	-	-
Male	0.84	0.68–1.04	0.115	0.84	0.67–1.05	0.120	0.77	0.56–1.06	0.106	0.74	0.53–1.04	0.079
**ART duration (years)**												
< 1	Ref	-	-	Ref	-	-	Ref	-	-	Ref	-	-
1–5	1.05	0.65–1.69	0.854	1.04	0.64–1.69	0.866	1.42	0.78–2.60	0.250	1.43	0.77–2.66	0.253
> 5	1.15	0.74–1.81	0.532	1.12	0.7–1.79	0.634	2.19	1.23–3.88	0.008	2.26	1.23–4.17	0.009
**ART regimen type**												
First-line	Ref	-	-	Ref	-	-	Ref	-	-	Ref	-	-
Second-line	0.61	0.34–1.09	0.095	0.69	0.36–1.30	0.249	0.38	0.19–0.72	0.003	0.49	0.23–1.05	0.066
**ART regimen backbone**												
Efavirenz or Nevirapine	Ref	-	-	Ref	-	-	Ref	-	-	Ref	-	-
Dolutegravir	1.27	0.96–1.68	0.095	1.24	0.93–1.67	0.148	1.35	0.90–2.04	0.150	1.31	0.84–2.03	0.234
Lopinavir or Ritonavir	0.85	0.56–1.27	0.420	0.92	0.59–1.43	0.700	0.68	0.40–1.17	0.163	0.70	0.38–1.29	0.255
**Sub-district**												
Ba-Phalaborwa	Ref	-	-	Ref	-	-	Ref	-	-	Ref	-	-
Greater Giyani	1.12	0.79–1.60	0.518	1.24	0.86–1.77	0.250	1.33	0.78–2.29	0.296	1.53	0.88–2.68	0.134
Greater Letaba	1.20	0.82–1.75	0.342	1.23	0.84–1.82	0.289	1.04	0.60–1.80	0.899	1.07	0.60–1.90	0.831
Greater Tzaneen	1.00	0.72–1.37	0.986	1.04	0.75–1.44	0.820	1.03	0.64–1.65	0.912	1.06	0.65–1.72	0.826
Maruleng	1.46	0.88–2.42	0.416	1.59	0.94–2.68	0.082	1.10	0.52–2.31	0.808	1.24	0.57–2.70	0.591

VL, viral load; ART, antiretroviral therapy; CI, Confidence Interval; DSD Model, Differentiated Service Delivery Model; Ref, reference group.

Similarly, at <1000 copies/mL, being enrolled on DSD model had increased odds of 12-month VS (AOR = 2.2; 95% CI: 1.4–3.5; *p* = 0.001) after adjusting for current age, gender, ART duration, ART regimen type, ART regimen backbone and sub-district. Being on ART over 5 years was also associated with increased odds of VS at < 1000 copies/mL (AOR = 2.3; 95% CI: 1.2–4.2; *p* = 0.009) (Table 5).

### Factors associated with 12-month viral suppression for differentiated service delivery group only (*N* = 646)

In multivariable logistic regression, there was no association between DSD model and 12-month VS (< 50 copies/mL and < 1000 copies) after adjusting for age at the last visit, gender, ART duration, ART regimen type, ART regimen backbone and sub-district (Online Appendix 1 Table 2-A1).

## Discussion

This study found that VS (< 50 copies/mL and < 1000 copies/mL) in adolescents in DSD was significantly higher than clinic-based care even in multivariable logistic analysis. However, ART retention was similar within DSD (92.7%) and clinic-based care (89.0%) for adolescents.

We found comparable retention in the DSD (92.7%) and clinic-based group (89.0%). In Mopani District, stable adolescents who are not eligible for DSD because of no valid identity document, for instance may receive multi-month dispensing at the clinic thereby enjoying similar benefits to those in facility-based DSD Models. This may contribute to the comparable retention in DSD and clinic-based care.

In our study, most adolescents (59%) accessed medications through facility pick-up points, while only 4% were in adherence clubs. Facility-based pick-up points are more common than external ones in rural areas because of the logistical challenges and limited infrastructure in the latter. In Mopani District, the scarcity of external pick-up points makes facility-based DSD the most accessible option. Starting in March 2020, during COVID-19 restrictions, there was a rapid shift from facility-based DSD to external pick-up points to reduce facility congestion and promote social distancing.^23^ However, post-COVID-19, many adherence clubs in the Mopani District were not reinstated. Data on adolescent preferences for DSD modalities are limited. A Ugandan study contrasts our data by reporting that adolescents preferred community-based client-led groups for their peer support.^24^

Within DSD models, facility pick-up points had the highest proportion of ART retention at 12 months (96.1%), although no association was found between DSD modality and 12-month retention in multivariable analysis. A South African study also showed higher loss to follow-up in community-based than clinic-based clubs among 18–24-year-olds.^25^

Adolescents with longer ART duration (1–5 years or > 5 years) had improved retention than those on ART for < 1 year. However, this finding may be biased, as in adolescents in long-term care on ART for > 1 year are likely to have remained adherent and remain engaged in care. In contrast, some studies report that longer treatment duration results in treatment fatigue and poor ART adherence among adolescents.^26,27,28^ Adolescents on dolutegravir-based regimens showed better retention than those on efavirenz or nevirapine-based regimens. This supports existing evidence that fewer side effects contribute to improved adherence. Clinic-based support groups may also improve ART knowledge and motivation. In this study, older adolescents (15–19 years) had lower 12-month retention than younger ones (10–14 years), possibly because of caregiver support for younger adolescents. This underscores the need for adolescent-friendly DSD models. Lower retention among adolescents aged 15–19 years was reported in South Africa and Namibia.^29,30^ In DSD models (*n* = 646), no significant association was found between DSD modality and 12-month retention after adjusting for covariates.

Overall VL coverage in this study was low, with only 70.7% having a VL. Other resource-limited settings have shown similar coverage, ranging from 54% to 68% in KwaZulu-Natal, South Africa, to 80.3% in Uganda.^31,32^ Barriers to adequate VL coverage include missing clinic appointments because of conflicting school commitments and caregivers collecting on behalf of the adolescents.^27^ While many Mopani District health facilities provide ART services, most do not offer laboratory services on weekends. To track the efficacy of ART, yearly VL testing is imperative. Synchronising prescription renewal with VL collection date and integrating VL collection at community and school health services may also improve VL coverage.

Viral suppression proportion at < 50 copies/mL cut-off was higher in DSD adolescents (63.5%), compared to clinic-based care (51.0%). Enrolment in DSD models was significantly associated with VS at < 50 copies/mL and < 1000 copies/mL after adjusting for confounders. At a higher VL threshold (< 400 copies/mL), data from six African countries reported VS ranging from 79% to 85% among clinically stable children and adolescents aged 0–19 years receiving multi-month dispensing.^33^ A similar pattern was observed for the < 1000 copies/mL threshold. A study from Malawi demonstrated that adolescents aged 10–19 years in teen clubs had better VS (cut-off < 1000 copies/mL) than standard clinical care.^34^ Another study in children and adolescents aged 2–18 years reported improved VS (< 1000 copies/mL) from 64% to 92% before and after implementing multi-month dispensing.^35^

Interestingly, despite a high proportion of suppression at < 1000 copies/mL, low-level viremia was common in 32.9% (449/1634) of the cohort as in other Southern African settings.^36^ The reasons for this are not clear, but most likely relate to suboptimal adherence.^37^

Within DSD Models, the proportion of VS (< 50 copies/mL) was highest within facility pick-up points (65.0%) and lowest within adherence clubs (52.4%). Adolescents at facility pick-up points also receive YCC services, including disclosure and adherence counselling, psychosocial support, sexual and reproductive healthcare, health talks, screening for sexually transmitted infections, tuberculosis and nutrition – these services were not offered in other modalities. This may contribute to enhanced adherence to ART, and consequently improved VS. As adherence clubs include members of different age groups, adherence support might not be adolescent-friendly.

This study had some strengths. This is the first study in South Africa to measure adolescent VS and retention in specific DSD models, such as facility pick-up points and external pick-up points. Moreover, this study contributes to the knowledge on the effectiveness of the DSD model in rural settings, specifically in Mopani District.

The study had several limitations. Because this is an observational study of routine programme data, the DSD and clinic-based groups are likely to be different at baseline. We found a higher proportion of older adolescents and those on ART for longer in DSD models. These adolescents are more likely to be eligible for DSD, have had additional opportunities for enrolment, and healthcare workers feel more comfortable enrolling them. We accounted for these factors in multivariate analysis, but it remains a challenge to evaluate outcomes of DSD in routine programmes. The sample size was small, as DSD models enrolled less than 30% of eligible adolescents in the district during the study period. Adherence clubs had too few participants to compare with other DSD models, preventing proportion difference testing. Only 12-month retention was reported; data beyond this period were unavailable, limiting long-term outcome analysis. Viral load collection was low, restricting the interpretation of VS results to adolescents with available VL results, potentially leading to an under- or over-estimation of VS proportions. Accurate retention data on DSD were unavailable on TIER.Net. The inclusion criteria for adolescents in clinic-based care did not consider high VL after initial suppression (< 50 copies/mL), only capturing a single point in time.

## Conclusion

This study highlights the role of DSD in improving VS and retention in care among adolescents. However, low VL coverage is a concern and may be improved by making ART services more accessible to adolescents and synchronising prescription renewal with VL sample collection. Furthermore, the persistence of low-level viremia warrants innovative adherence support without adding to the visit burden.
